# The Impact of Prolonged Dental Chair Utilization on Cervical Spine Mobility and Pain among Dental Internship Students: A Cross-Sectional Observational Study from the United Arab Emirates.

**DOI:** 10.12688/f1000research.182694.1

**Published:** 2026-06-16

**Authors:** Mariam Hadi Elabed, Hesham Jarbou, Noor Alsalim, Animesh Hazari, Praveen Kumar Kandakurti, Mamdouh Gabr

**Affiliations:** 1 College of Health Sciences, Gulf Medical University, Ajman, United Arab Emirates, United Arab Emirates

**Keywords:** Dentistry; Cervical range of motion; Neck pain; Ergonomics; Musculoskeletal disorders.

## Abstract

**Background:**

Dentistry is considered a high-risk profession for developing work-related musculoskeletal disorders (MSDs), particularly in the cervical region, due to prolonged static postures, repetitive movements, and sustained neck flexion during clinical procedures. Limited evidence exists regarding the early short-term effects of prolonged dental chair utilization on cervical spine mobility among dental interns.

**Objective:**

To evaluate changes in cervical spine range of motion (ROM) and neck pain associated with prolonged dental chair utilization among dental internship students **.**

**Methods:**

A prospective short-term observational repeated-measures study was conducted among 40 dental internship students at Thumbay Dental Hospital, Gulf Medical University, Ajman, United Arab Emirates. Cervical ROM was assessed using a validated digital inclinometer for flexion, extension, bilateral lateral flexion, and bilateral rotation. Neck pain intensity was evaluated using the Visual Analogue Scale (VAS). Assessments were performed at baseline and repeated after one month of routine clinical exposure involving prolonged dental chair utilization. Paired-sample t-tests were used for statistical analysis, with significance set at p ≤ 0.05.

**Results:**

A statistically significant increase in neck pain intensity was observed following one month of clinical exposure (p < 0.05). Cervical ROM measures demonstrated no significant differences in flexion, extension, left rotation, or bilateral lateral flexion between baseline and follow-up assessments. However, right cervical rotation showed a statistically significant increase at follow-up. These findings suggest that short-term occupational exposure primarily affects pain perception before substantial mobility impairment develops.

**Conclusion:**

Prolonged dental chair utilization during internship training significantly increased cervical pain among dental interns, while cervical mobility remained largely preserved over the short term. Early ergonomic interventions, posture education, and preventive physiotherapy strategies should be incorporated into dental curricula to reduce occupational musculoskeletal risks.

**Trial Registration Status**: Not Applicable.

## Introduction

Dentistry is widely recognized as an occupation associated with a high risk of work-related musculoskeletal disorders (MSDs), largely due to prolonged static postures, repetitive movements, and suboptimal ergonomic conditions. ^1^ Dental professionals frequently maintain sustained neck flexion and rotation to obtain adequate visualization of the operative field, placing considerable biomechanical stress on the cervical spine. ^2^ Evidence suggests that the cervical region is among the most commonly affected anatomical areas, with symptoms often beginning during undergraduate clinical training. ^2, 3^

Dental internship programs typically require interns to work for extended hours in constrained postures, potentially accelerating the onset of cervical pain and functional limitations. ^4^ In the United Arab Emirates, dental interns undergo a 12-month internship involving approximately 6–7 clinical hours per day, which may increase cumulative cervical loading. ^5^ While previous studies have documented the prevalence of neck pain among dentists and dental students, ^6, 7^ limited data exist regarding the early impact of prolonged dental chair use on cervical spine mobility during internship training. Therefore, this study aimed to evaluate changes in cervical ROM and neck pain associated with prolonged dental chair utilization among dental internship students.

## Materials and methods

### Study design

A prospective short-term observational repeated-measures study was conducted to investigate changes in cervical spine mobility and neck pain associated with prolonged dental chair utilization among dental internship students. The repeated-measures design enabled assessment of within-subject changes over time following routine clinical exposure.

### Study setting

The study was conducted at Thumbay Dental Hospital, affiliated with Gulf Medical University, Ajman, United Arab Emirates, during the official internship training period. The hospital serves as a major teaching and clinical facility where dental interns perform comprehensive patient management under clinical supervision.

### Ethical considerations

Ethical approval for the study was obtained from the Institutional Ethics Committee of Gulf Medical University prior to commencement of data collection (IRB-COHS-STD-103-APR-2024). All study procedures were performed in accordance with the ethical principles outlined in the Declaration of Helsinki for research involving human participants. Participants were informed about the study objectives, procedures, potential risks, and voluntary nature of participation. Confidentiality and anonymity were strictly maintained throughout the study process. Written informed consent was obtained from all participants before enrollment.

### Participants

Dental internship students were recruited using convenience sampling from the internship program at Thumbay Dental Hospital. Participants included male and female dental interns aged between 22 and 24 years who were actively engaged in clinical duties during the study period.

### Inclusion criteria

Participants were included if they:

Were dental fresh graduate internship students, both genders.

Were right-hand dominant.

Were actively participating in routine clinical duties.

Agreed to participate voluntarily and provided written informed consent.

Right-handed participants were specifically selected to minimize variability associated with operator positioning and dental chair orientation during clinical procedures.

### Exclusion criteria

Participants were excluded if they had:

Congenital or acquired cervical spine deformities.

Diagnosed musculoskeletal or neuromuscular disorders.

Previous cervical trauma or spinal surgery.

Current pregnancy.

Acute cervical injury or inflammatory conditions.

Refusal to participate or inability to complete study procedures.

### Sample size

A total of 40 dental interns participated in the study. Due to the exploratory nature of the investigation and the limited availability of comparable pilot data addressing short-term cervical mobility changes among dental interns, a formal a priori sample size estimation was not feasible. Nevertheless, the selected sample size was considered appropriate for preliminary evaluation of repeated-measures outcomes and for generating foundational data to support future larger-scale investigations.

### Outcome measures

Cervical spine range of motion (ROM) was assessed for flexion, extension, right and left lateral flexion, and right and left rotation using a validated digital inclinometer. ^8^ Neck pain intensity was measured using the Visual Analogue Scale (VAS), a reliable and widely used tool for pain assessment. ^9^ Participants were assessed in a standardized seated position with feet supported on the floor and the thoracic spine maintained in neutral alignment. Instructions and testing procedures followed published reliability protocols to ensure consistency across measurements. Three trials were obtained for each movement direction, and the mean value was used for analysis.

### Data collection procedure

Eligible participants received detailed verbal and written explanations regarding the study procedures before signing informed consent forms. Baseline demographic information, including age, sex, and dominant hand, was collected electronically. Baseline cervical ROM measurements and pain assessments were performed before initiation of the observational period. All measurements were conducted under standardized environmental conditions by the same examiner to minimize inter-rater variability. Participants were instructed to avoid vigorous physical activity immediately before testing. Following baseline assessment, participants continued their routine internship clinical activities involving prolonged dental chair utilization and sustained clinical postures for approximately one month. Clinical duties typically involved 6–7 hours of patient-related activities per day, including restorative procedures, oral examinations, scaling, and other operative tasks requiring prolonged visual concentration and static postural maintenance.

At the end of the one-month observational period, cervical ROM and VAS pain assessments were repeated using identical testing procedures and positioning protocols.

### Reliability of measurements

The digital inclinometer used for cervical ROM assessment has demonstrated excellent intra-rater and inter-rater reliability in previous studies, with intraclass correlation coefficient (ICC) values ranging from 0.80 to 0.95 for cervical movements. Standardized participant positioning and repeated measurement procedures were employed in the present study to enhance measurement consistency and minimize examiner-related variability.

### Statistical analysis

Data analysis was performed using Statistical Package for the Social Sciences (SPSS) software. Descriptive statistics were calculated for all demographic and outcome variables. Continuous variables were expressed as mean ± standard deviation. Data normality was assessed using the Shapiro–Wilk test before inferential analysis. Paired-sample t-tests were used to compare baseline and follow-up values for cervical ROM and pain intensity. Statistical significance was established at p ≤ 0.05. In addition to statistical significance testing, effect sizes (Cohen’s d) and 95% confidence intervals were calculated to determine the magnitude and precision of observed changes. Cohen’s d values were interpreted as small (0.2), moderate (0.5), and large (0.8) effects.

## Results

The study included 40 dental internship students from Thumbay Dental Hospital, Ajman, United Arab Emirates. Demographic characteristics of the participants are presented in Table 1.

**Table 1.** Demographic data of the participants.

Variables		N* (%)
**Gender**	Male	13 (32.5%)
	Female	27 (67.5%)
**Age**	23	28 (70%)
	24	12 (30%)

Comparison of cervical ROM between baseline and follow-up measurements demonstrated no statistically significant differences in cervical flexion, extension, left rotation, or bilateral lateral flexion following one month of routine dental clinical exposure. However, a statistically significant increase in right cervical rotation ROM was observed at follow-up assessment ( Table 2).

**Table 2.** Comparison between mean values of the first and second measurement of cervical range of motion.

	First	Second	
	Mean ± SD	Mean ± SD	P-value
**Flexion**	54.13 ± 8.4	54.28 ± 7.6	.943
**Extension**	54.90 ± 9.9	55.48 ± 9.8	.502
**Right lateral flexion**	38.82 ± 8.5	40.62 ± 7.7	.179

(CROM = cervical range of motion)

Pain assessment using the VAS revealed a statistically significant increase in neck pain intensity following the one-month clinical exposure period. These findings indicate that prolonged dental chair utilization during internship training was associated with increased cervical discomfort despite relatively preserved cervical mobility.

**Figure 1.**
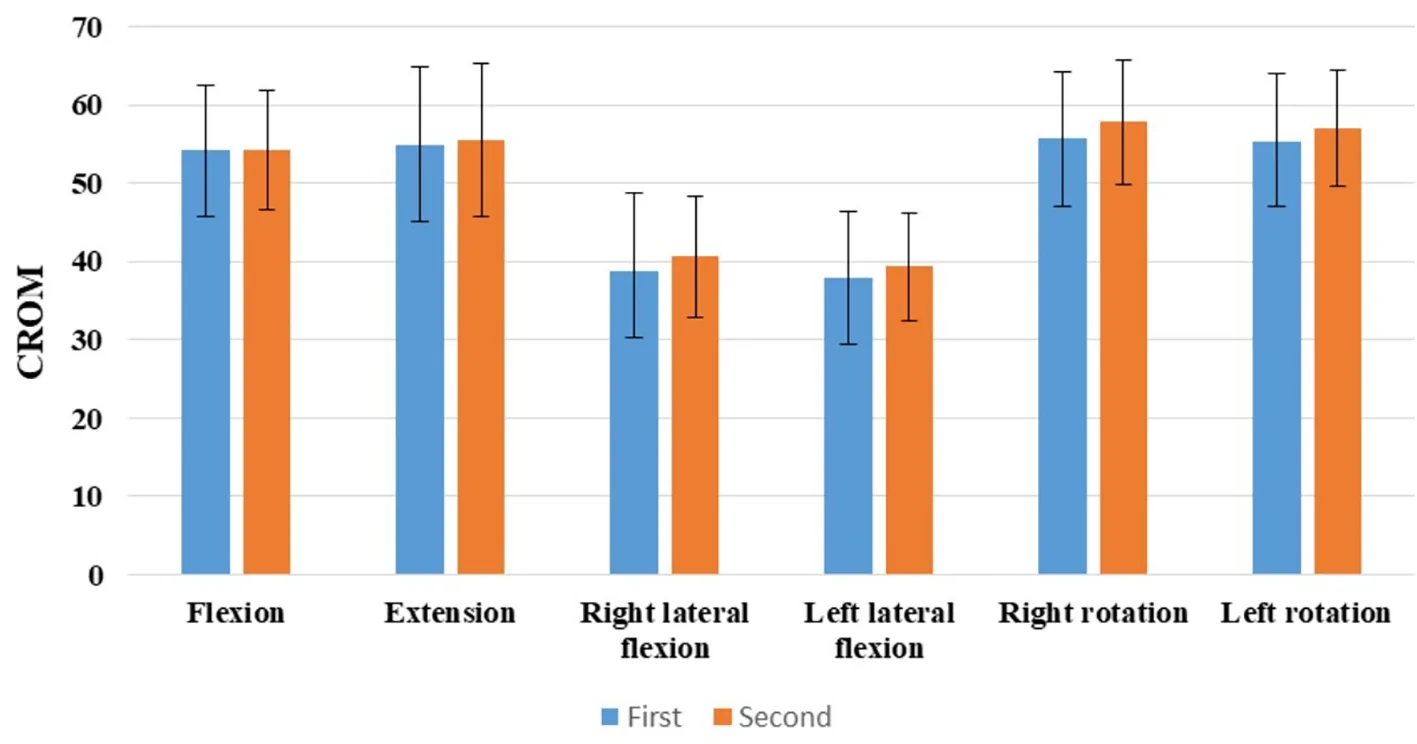
Comparison between the mean values of the first and second measurements of cervical range of motion.

**Figure 2.**
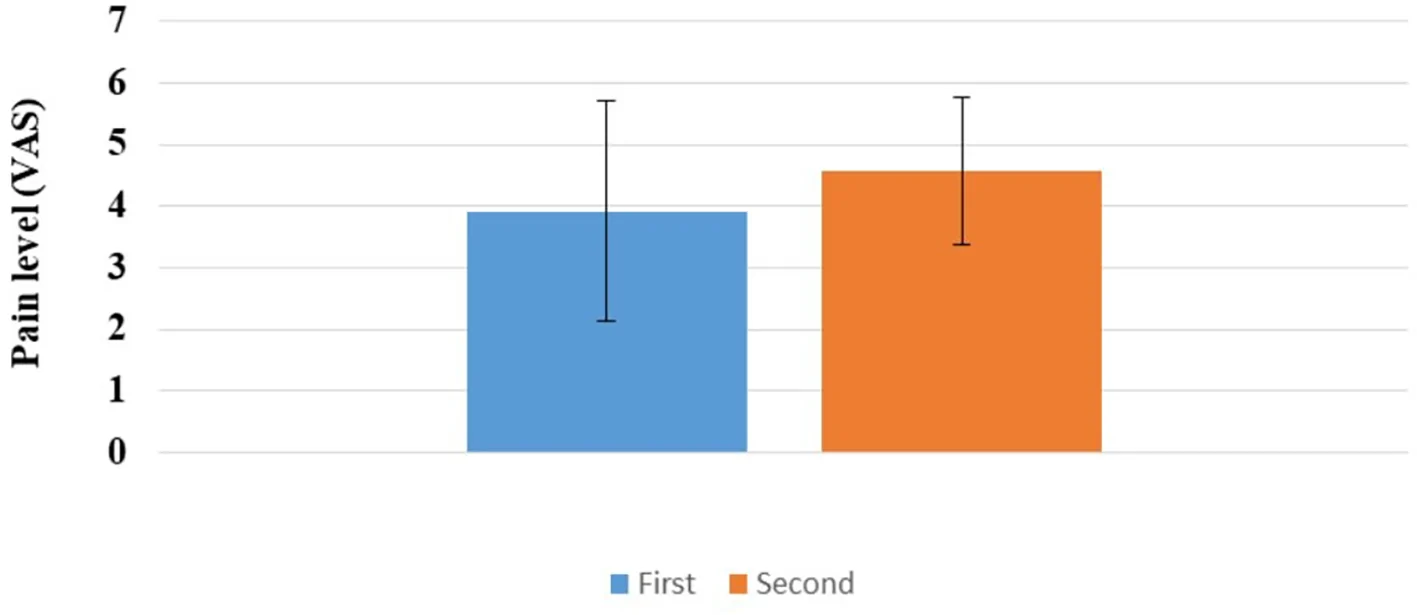
Comparison between mean values of cervical pain level in the first and second measurement.

Figure 1 illustrates the comparison between baseline and follow-up cervical ROM measurements, while Figure 2 presents the comparison of neck pain intensity values between the two assessment periods. The results in Figure 1 indicated no significant changes in flexion, extension, right lateral flexion, or left lateral flexion. However, a significant change was observed in right rotation (p < 0.001), while left rotation showed no significant change (p = 0.076). A significant increase in pain levels was seen over time, with mean VAS scores rising from 3.92 ± 1.873 initially to 4.58 ± 1.259 (p < 0.001) ( Figure 2).

Inferential analysis demonstrated statistically significant increases in right cervical rotation (mean difference = 2.15°, 95% CI: 0.12 to 4.18, p = 0.038) and VAS pain scores (mean difference = 0.66, 95% CI: 0.08 to 1.24, p = 0.027) following one month of clinical exposure. No statistically significant differences were observed for cervical flexion, extension, bilateral lateral flexion, or left cervical rotation (p > 0.05) ( Table 3).

**Table 3.** Comparison of cervical ROM and pain scores between baseline and follow-up assessments (n = 40).

Variable	Baseline Mean ± SD	Follow-up Mean ± SD	Mean Difference	95% CI	Cohen’s d	Exact p- value
Cervical Flexion (°)	54.13 ± 8.45	54.28 ± 7.70	+0.15	−3.42 to 3.72	0.02	0.931
Cervical Extension (°)	54.90 ± 9.94	55.48 ± 9.87	+0.58	−3.82 to 4.98	0.06	0.794
Right Lateral Flexion (°)	38.82 ± 8.57	40.62 ± 7.77	+1.80	−1.83 to 5.43	0.22	0.327
Left Lateral Flexion (°)	37.95 ± 8.63	39.33 ± 6.82	+1.38	−2.05 to 4.81	0.18	0.425
Right Rotation (°)	55.65 ± 8.69	57.80 ± 7.95	+2.15	0.12 to 4.18	0.26	0.038
Left Rotation (°)	55.32 ± 8.36	56.95 ± 7.46	+1.63	−1.57 to 4.83	0.21	0.311
VAS Pain Score	3.92 ± 1.87	4.58 ± 1.26	+0.66	0.08 to 1.24	0.42	0.027

## Discussion

The present study evaluated the short-term effects of prolonged dental chair utilization on cervical spine mobility and pain among dental internship students. The principal findings indicate a significant increase in cervical pain over time, while cervical range of motion remained largely unchanged, except for a significant increase in right cervical rotation.

The absence of significant changes in cervical flexion, extension, and lateral flexion suggests that short-term exposure to prolonged static dental postures may not immediately compromise gross cervical mobility in young adults. This finding contrasts with reports of reduced cervical ROM among experienced dental professionals, suggesting that cumulative exposure over longer durations may be required for measurable mobility impairments to develop. ^10, 11^ The relatively short follow-up period and the adaptive capacity of younger musculoskeletal systems may partly explain these findings.

A statistically significant increase in right cervical rotation was observed. This may reflect adaptive or compensatory movement strategies adopted by interns to accommodate visual and ergonomic demands during dental procedures. Right-handed dental practitioners frequently assume asymmetric postures involving sustained rotation and side bending to optimize visibility within the oral cavity. ^12^ Repetitive use of these postures may promote directional mobility adaptations, resulting in increased range in the dominant direction of movement.

Despite minimal changes in cervical mobility, a significant increase in neck pain intensity was observed over the one-month period. Prolonged static postures are known to impair muscle perfusion, reduce oxygen delivery, and promote accumulation of metabolic by-products, leading to muscle fatigue and pain. ^13^ Sustained low-level muscle contractions can also compress vascular structures and joint tissues, contributing to ischemia and nociceptive sensitization. ^14^ These mechanisms may explain the observed rise in pain scores despite preserved joint mobility.

The findings align with existing literature documenting a high prevalence of neck pain among dental professionals and students, ^6^ even in the absence of marked structural or functional impairments. Early onset of pain during internship training highlights the importance of addressing ergonomic risk factors at the beginning of professional practice. Without intervention, persistent pain may progress to chronic musculoskeletal disorders, reduced work capacity, and diminished quality of life.

The findings of the current study are consistent with recent systematic reviews reporting high rates of cervical pain among dental professionals and students exposed to prolonged static postures and repetitive occupational demands. A recent systematic review by Ehsan Rafeemanesh identified poor cervical posture, prolonged working duration, inadequate rest intervals, and increased patient load as major contributors to neck pain among dentists. ^15^ Similarly, another systematic review demonstrated that sustained forward head posture and asymmetric trunk positioning substantially increase cervical muscular loading and cumulative trauma exposure among dental practitioners. ^1^

The present findings also align with evidence suggesting that musculoskeletal pain may emerge before measurable functional limitation develops. Young clinicians may initially compensate through adaptive neuromuscular strategies and preserved tissue flexibility, thereby maintaining cervical ROM despite increasing pain symptoms. This may explain why cervical mobility remained largely unaffected in the present study despite significant increases in pain intensity. Recent ergonomic intervention reviews have additionally emphasized the protective role of stretching exercises, ergonomic education, indirect visualization techniques, and scheduled rest breaks in reducing occupational cervical strain among dental professionals. ^16^ These findings support the integration of preventive ergonomic training during undergraduate and internship dental education.

Moreover, recent occupational health literature has highlighted that early-stage cervical symptoms among dental interns may progress into chronic work-related musculoskeletal disorders if preventive strategies are not implemented promptly. ^17^ The current study therefore contributes important preliminary evidence regarding the early onset of occupational cervical pain during internship training. Practical ergonomic challenges experienced by dental professionals have also been reflected in contemporary professional discussions emphasizing the importance of appropriate patient positioning, neutral posture maintenance, and avoidance of excessive cervical flexion during clinical procedures.

### Limitations

Several limitations should be considered when interpreting the findings of this study. First, the relatively small sample size and recruitment from a single institution may limit the generalizability of the results to broader populations of dental interns. Second, the predominance of female participants may have introduced potential gender-related variability in pain perception and musculoskeletal response. Third, the short follow-up duration limited evaluation of long-term cervical adaptations associated with prolonged occupational exposure. Longitudinal investigations are needed to determine whether persistent exposure eventually results in measurable mobility restrictions or chronic musculoskeletal dysfunction. Additionally, ergonomic variables such as working posture, patient positioning, type of dental procedures performed, psychosocial stress, and physical activity levels were not objectively assessed. Inclusion of posture analysis tools, electromyographic assessment, and ergonomic risk scoring systems in future studies may provide more comprehensive understanding of the mechanisms underlying cervical symptoms in dental professionals.

### Clinical and educational implications

The findings underscore the need for early ergonomic education, posture training, and preventive physiotherapy interventions during dental internship programs. Incorporating ergonomic awareness and regular movement breaks into clinical training may help mitigate the development of cervical pain and long-term musculoskeletal disorders.

### Future research

Future studies should employ longitudinal designs with larger, multi-center cohorts to evaluate long-term changes in cervical mobility and pain. Integration of ergonomic risk assessment tools and objective posture analysis may further enhance understanding of occupational risk factors in dentistry.

## Conclusion

Prolonged dental chair utilization during internship training was associated with a significant increase in neck pain among dental interns, while cervical spine mobility remained largely preserved over the short-term observational period. The findings suggest that pain symptoms may develop before measurable functional mobility impairments become apparent. Early implementation of ergonomic education, posture correction strategies, and preventive physiotherapy interventions within dental training programs is strongly recommended to minimize occupational cervical stress and reduce the future burden of musculoskeletal disorders among dental professionals.

## Ethics approval and consent to participate

The study was conducted in line with the Declaration of Helsinki for human participants and approved by the Institutional Ethics Committee, Gulf Medical University, Ajman, United Arab Emirates (IRB-COHS-STD-103-APR-2024).

## Data Availability

The complete dataset has been given in the online repository. [Figshare]: [DENTAL CHAIR AND MOBILITY]. [ https://doi.org/10.6084/m9.figshare.32287353] https://figshare.com/articles/dataset/DENTAL_CHAIR_AND_MOBILITY/32287353. ^18^ Licence CC4.0. The project contains the following underlying data: [Analysed data, IRB Approval].
