# Supplementary material for: The Impact of Prolonged Dental Chair Utilization on Cervical Spine Mobility and Pain among Dental Internship Students: A Cross-Sectional Observational Study from the United Arab Emirates
Source: F1000Res. 2026 Jun 16;15:948. [Version 1] doi: 10.12688/f1000research.182694.1 (PMC13563008; doi:10.12688/f1000research.182694.1)
Supplement: Peer review history

# Reviewer report: Reviewer response for version 1

**DOI:** 10.5256/f1000research.201658.r515172

**Published:** 2026-09-02

**Authors:** Rashmi Venkatesh

**Affiliations:** 1 K. M. Shah Dental College and Hospital, Sumandeep Vidyapeeth (Deemed to be University), Vadodara, Gujarat, India

The article provides valuable preliminary evidence on short-term cervical pain development in dental interns. Article is well-written, structured, and accessible. Article clearly emphasizes early ergonomic intervention. Thoughtful interpretation of increased pain despite preserved mobility. Authors have linked findings to ergonomic demands and compensatory strategies very well also integrated relevant systematic reviews and occupational health literature to strenghten evidence. 

**Recommendation:** **Minor revisions**—strengthen the discussion of clinical significance, expand on limitations, and sharpen the articulation of the research gap.

Is the work clearly and accurately presented and does it cite the current literature?

Yes

If applicable, is the statistical analysis and its interpretation appropriate?

Yes

Are all the source data underlying the results available to ensure full reproducibility?

Yes

Is the study design appropriate and is the work technically sound?

Yes

Are the conclusions drawn adequately supported by the results?

Yes

Are sufficient details of methods and analysis provided to allow replication by others?

Yes

Reviewer Expertise:

Dentistry, Oral Medciine and Radiology, Oral Diseases, Forensic Odontology

I confirm that I have read this submission and believe that I have an appropriate level of expertise to confirm that it is of an acceptable scientific standard.

# Reviewer report: Reviewer response for version 1

**DOI:** 10.5256/f1000research.201658.r495903

**Published:** 2026-06-30

**Authors:** Tamer Shousha

**Affiliations:** 1 University of Sharjah, Sharjah, United Arab Emirates; 2 Center of Excellence for Healthy Ageing, University of Sharjah (Ringgold ID: 59105), Sharjah, Sharjah, United Arab Emirates; 3 Ad Doqi, Cairo University, Faculty of Physical Therapy (Ringgold ID: 531226), Giza Governorate, Egypt

The manuscript is generally well organized, follows a logical structure, and addresses a clinically meaningful research question. The conclusions are partially supported by the data; however, several methodological weaknesses, statistical inconsistencies, and issues related to study design reduce confidence in the findings. In particular, the terminology used to describe the study design, the absence of exposure quantification, the lack of control for important confounding variables, and several inconsistencies in statistical reporting require careful revision.

**Major Comments**

**1. Inconsistency in Study Design**

 The manuscript title describes the study as a "Cross-Sectional Observational Study," whereas the Methods clearly describe a prospective repeated-measures observational design with baseline and one-month follow-up assessments.

 These two study designs are fundamentally different.

 A cross-sectional study involves measurements obtained at a single time point, whereas the present investigation evaluates within-subject changes over time.

**2. Exposure Measurement is Poorly Defined**

 The primary exposure is described simply as "prolonged dental chair utilization."

 However, no quantitative exposure variables were collected, such as:

- actual chair time/day
- number of patients treated
- duration of procedures etc...

**3. Lack of Control for Important Confounding Variables**

 Neck pain among dental professionals is multifactorial.

 Several important variables were not measured, including:

- physical activity level
- previous exercise habits
- psychosocial stress
- sleep quality
- computer use outside clinical hours
- previous episodes of neck pain / pathology

These factors may substantially influence both pain and cervical mobility.

**4. Sample Size Justification is Weak**

 The authors state that a formal sample size calculation was not feasible because of limited pilot data.

**5. Clinical Significance is Not Adequately Discussed**

 The manuscript focuses exclusively on statistical significance without discussing:

- Minimal Clinically Important Difference (MCID) or
- Minimal Detectable Change (MDC) or providing clinical interpretation

**Statistical Comments**

**Confidence Intervals**

 Several confidence intervals reported in Table 3 appear inconsistent with the observed mean differences.

 For example:

 Flexion:

 Mean difference = +0.15°

 95% CI = −3.42 to +3.72°

**Discussion**

 The discussion is generally balanced and well referenced.

 However, several statements imply causation:

 "Prolonged dental chair utilization caused cervical pain."

 Strictly speaking, the study demonstrates an association over one month rather than definitive causation.

**Limitations Requiring Further Discussion**

 The current limitations section is appropriate but incomplete. Additional limitations should include:

- absence of a control group
- lack of exposure quantification
- absence of assessor blinding

Is the work clearly and accurately presented and does it cite the current literature?

Yes

If applicable, is the statistical analysis and its interpretation appropriate?

Partly

Are all the source data underlying the results available to ensure full reproducibility?

Yes

Is the study design appropriate and is the work technically sound?

Partly

Are the conclusions drawn adequately supported by the results?

Yes

Are sufficient details of methods and analysis provided to allow replication by others?

Partly

Reviewer Expertise:

Physiotherapy and rehabilitation of the Musculoskeletal system, sport injuries, spine rehab., TMJ dysfunction, Ageing and longevity, physical activity.

I confirm that I have read this submission and believe that I have an appropriate level of expertise to confirm that it is of an acceptable scientific standard.
